# *Abiotrophia defectiva* and *Granulicatella*: A Literature Review on Prosthetic Joint Infection and a Case Report on *A. defectiva* PJI and Concurrent Native Valve Endocarditis

**DOI:** 10.3390/microorganisms13051113

**Published:** 2025-05-12

**Authors:** Cristina Seguiti, Edda Piacentini, Angelica Fraghì, Mattia Zappa, Elia Croce, Angelo Meloni, Marco Cirillo, Clarissa Ferrari, Chiara Zani, David Belli, Tony Sabatini, Paolo Colombini

**Affiliations:** 1 SS Malattie Infettive, Fondazione Poliambulanza Istituto Ospedaliero, 25124 Brescia, Italy; 2 UOC Medicina Generale e Geriatria, Fondazione Poliambulanza Istituto Ospedaliero, 25124 Brescia, Italy; 3 Medicina di Laboratorio, Fondazione Poliambulanza Istituto Ospedaliero, 25124 Brescia, Italy; 4 UOC Medicina Interna, Policlinico Universitario Agostino Gemelli IRCCS, 00168 Roma, Italy; 5 Direzione Sanitaria, Fondazione Poliambulanza Istituto Ospedaliero, 25124 Brescia, Italy; 6 UOC Cardiochirurgia, Fondazione Poliambulanza Istituto Ospedaliero, 25124 Brescia, Italy; 7 Research and Clinical Trials Unit, Fondazione Poliambulanza Istituto Ospedaliero, 25124 Brescia, Italy; clarissa.ferrari@poliambulanza.it (C.F.);

**Keywords:** *Abiotrophia defectiva*, *Granulicatella*, prosthetic joint infection

## Abstract

Together with *Granulicatella* spp., *A. defectiva* was formerly classified within the group of nutritionally variant streptococci (NVS). NVS-related endocarditis has been associated with higher rates of complications, bacteriological failure, and mortality compared to other streptococci, partially due to challenges related to timely and accurate identification. PJI caused by *A. defectiva* are rarely reported, and standardized management strategies have not yet been established. We describe a case of a 68-year-old man with concomitant *A. defectiva* PJI and native mitral valve endocarditis. The patient was managed conservatively for endocarditis and subsequently underwent a two-stage arthroplasty of the infected prosthetic knee. *A. defectiva* was identified using MALDI-TOF mass spectrometry on both synovial fluid and blood cultures. As penicillin susceptibility data were not available, the patient was treated with vancomycin at a dose of 2 g/day, resulting in a favorable clinical response. In addition, we performed a literature review on *A. defectiva* and *Granulicatella* PJI. Despite the limited number of reported cases in the literature, the findings suggest a potential correlation between clinical outcomes and antimicrobial treatment duration. Further comprehensive studies are needed to establish standardized management strategies for *A. defectiva* and *Granulicatella* PJI.

## 1. Introduction

*Abiotrophia defectiva* is commonly found as a commensal in the oral, urogenital, and intestinal microbiota. Despite its typically benign presence, it may express virulence factors that confer pathogenic potential. Although infections are infrequent, *A. defectiva* has been recognized as a possible causative agent of bacteremia and culture-negative endocarditis [1,2].

Formerly classified within the nutritionally variant streptococci (NVS) group, along with *Granulicatella* species, these bacteria are characterized as fastidious Gram-positive organisms that require enriched media to grow under standard culture conditions. Consequently, their isolation and subsequent antimicrobial susceptibility testing can pose significant challenges for clinical microbiology laboratories [3]. Proteomic analyses have further demonstrated that these organisms express several multifunctional “moonlighting proteins”, which contribute to enhanced bacterial colonization, the stimulation of inflammatory responses, the disruption of host cellular homeostasis, and the modulation of host immunity [1]. *A. defectiva* exhibits a marked ability to adhere to fibronectin, which accounts for its tropism for endothelial valvular tissue. Although the initial clinical presentation may be indolent, disease progression with deterioration is commonly observed, characterized by the formation of large vegetations and significant structural changes to the affected valves. These pathological changes can lead to severe embolic events and progressive heart failure [4].

Osteomyelitis and osteoarticular infections caused by *A. defectiva* have rarely been reported. Nevertheless, there is growing interest in the pathogenic potential of both *Granulicatella* and *Abiotrophia* species in implant-associated infections. The concomitant occurrence and management of endocarditis and prosthetic joint infection (PJI) has been reported for *Granulicatella para-adiacens*, with favorable outcomes achieved using the DAIR approach for PJI and conservative treatment for endocarditis [5], but not for *A. defectiva*.

Determining the most effective antimicrobial therapy for NVS infections remains challenging. The inherently slow replication rate of these organisms may reduce their susceptibility to penicillin and contribute to the development of penicillin tolerance. Among available agents, ceftriaxone demonstrates the highest efficacy; however, its activity against biofilm-associated bacteria remains limited. In their analysis of susceptibility profiles to newer antimicrobials, Cañas et al. reported the emergence of high-level resistance to daptomycin. Notably, daptomycin nonsusceptibility may be present at baseline or may develop during the course of therapy, whether administered as monotherapy or in combination [6].

We conducted a review of the existing literature on prosthetic joint infections caused by *A. defectiva* and *Granulicatella* species, supplemented by a clinical case of knee PJI with concurrent endocarditis due to *A. defectiva*.

## 2. Case Description

A 68-year-old male with a medical history of insulin-dependent type 2 diabetes mellitus and mitral valve Barlow syndrome underwent total left knee arthroplasty in October 2023 for painful osteoarthritis at an outside institution. Notably, he had experienced a self-limiting febrile episode prior to the prosthetic implantation. Approximately one month postoperatively, the patient developed another febrile illness, initially attributed to a urinary tract infection, with *Enterococcus faecalis* isolated from a urine culture. Despite receiving oral antimicrobial therapy and the resolution of urinary symptoms, the patient continued to experience intermittent fever and developed initial mild knee swelling without associated joint pain. An initial arthrocentesis revealed Gram-positive cocci in the synovial fluid, although subsequent cultures remained negative.

The patient was referred to our hospital in January 2024 for further evaluation. Repeat blood cultures and synovial fluid analysis were performed. The synovial fluid appeared turbid, with elevated glucose levels (164 mg/dL) and a leukocyte count of 16,500 cells/mm^3^ with 85% neutrophils. Both blood and synovial fluid cultures yielded the growth of *A. defectiva*, identified via MALDI-TOF mass spectrometry.

Transoesophageal echocardiography demonstrated multiple large vegetations on the mitral valve (6 × 3 mm, 13 × 7 mm, and 6 × 4 mm) with severe mitral regurgitation despite the absence of clinical signs of heart failure. Following cardiothoracic surgery consultation, a conservative management strategy was adopted, considering the patient’s hemodynamic stability and the associated risk–benefit profile. Due to unavailability of antimicrobial susceptibility testing, the patient was initiated on vancomycin (2 g/day), resulting in prompt defervescence and a decline in C-reactive protein levels. Despite clinical improvement, radiological imaging identified multiple asymptomatic embolic events in the spleen and brain.

The patient was referred to the original institution for a two-stage arthroplasty, which was performed 4 weeks after the initiation of antibiotic therapy. Antimicrobial treatment was continued for six weeks following prosthesis removal. The outpatient follow-up demonstrated normalization of inflammatory biomarkers, and the patient remains under regular cardiology and cardiothoracic follow-up to evaluate the potential need for mitral valve surgery. He underwent successful prosthetic joint reimplantation in November 2024 and, to date, remains free of infection relapse.

## 3. Literature Review Results

We conducted a literature search on Pubmed and Google Scholar by using the following research string: ((*Abiotrophia*) OR (*Granulicatella*)) AND (prosthetic joint infection). Table A1 and Table A2 in Appendix A present the characteristics of the clinical cases. A total of 21 case reports were found; we analyzed data from 22 patients (including our own case) to explore potential associations between clinical factors and outcome. Considering all cases, we aimed to identify variables associated with the dichotomous outcome classified as cured vs. non-cured PJI, defining “non-cured” as reported relapse, reinfection, or complicated clinical course. Although the small sample size precluded statistically robust conclusions, some interesting patterns have been highlighted (Table A3, Appendix A). A total of 11 cases involving *A. defectiva* and 10 cases of *Granulicatella adiacens* were analyzed, and we only analyze 1 case of *Granulicatella para-adiacens*. Overall, the outcomes were favorable in most patients treated for *A. defectiva* or *Granulicatella*-related PJI: one patient experienced a true relapse, one died due to pneumonia, and another developed a reinfection with *Enterobacter cloacae*, which complicated the clinical course. The mean age of the cured patients was 66.9 (SD = 7.87), approximately 5 years younger than the non-cured group. Interestingly, the surgical strategy appeared to be a potential factor influencing favorable outcomes: two out of the three non-cured patients were managed with a debridement, antibiotics, and implant retention (DAIR) approach, whereas the majority of cured patients (73.7%) underwent a two-stage exchange procedure. However, given that some cases were successfully treated with the DAIR approach, the association between surgical strategy and outcome may depend more on the appropriateness of patient selection and adherence to established indications for DAIR. Features related to treatment duration appeared to be of particular interest. Despite the limited number of cases, this variable reached statistical significance: the average duration of treatment in the non-cured group was 5.7 vs. 15.2 weeks in the cured group (*p* = 0.048). These findings suggest potential prognostic factors associated with PJI outcomes, which warrant confirmation in larger, dedicated studies.

## 4. Discussion

Formerly assigned to the NVS group, *A. defectiva* (along with *Granulicatella* spp.) is more commonly reported in the literature as a causative agent of bacteremia and endocarditis rather than prosthetic joint infections [7]. *A. defectiva* typically colonizes the skin and mucosal surfaces, with odontogenic procedures or other invasive or micro-invasive procedures often serving as a primary source of bacteremia. Oral colonization rates have been reported to reach approximately 12%, and NVS can also colonize the gastrointestinal and genitourinary tracts [8].

Their fastidious growth requirements often result in misidentification and underdiagnosis, thereby underestimating their pathogenic role and impacting decisions regarding de-escalation and antimicrobial stewardship. As noted by Téllez et al., *A. defectiva* and *Granulicatella* share similar clinical characteristics and outcomes, resembling other forms of subacute infective endocarditis in terms of mortality rates [9]. Nevertheless, several studies have reported higher complication rates, including embolism, valvular and peri-valvular damage, and antibiotic treatment failure, even when penicillin susceptibility is documented [10]. These complications are believed to be associated with the microorganisms’ ability to produce exopolysaccharide in vivo and their strong affinity for fibronectin binding. In addition, NVS species have significantly slower generation times compared to viridans streptococci, which may reduce the efficacy of beta-lactam antibiotics and contribute to the mechanisms of penicillin tolerance [11]. Standard antimicrobial susceptibility testing is often not feasible due to poor growth; in such cases, molecular diagnostic methods such as 16S rRNA sequencing are frequently necessary for microbial identification. Ratcliffe et al. [12] compared identification methods, demonstrating that Vitek 2 is less effective than MALDI-TOF mass spectrometry in detecting these fastidious organisms. Although 16S rRNA gene sequencing is considered the gold standard for identifying difficult-to-culture microorganisms, its use remains limited by high costs and prolonged turnaround times [12].

Prosthetic joint infections caused by *A. defectiva* are rarely reported in the literature, and no specific treatment guidelines exist for managing infections involving prosthetic hardware. In the context of infective endocarditis, the American Heart Association guidelines recommend treatment based on penicillin MIC for *Granulicatella* and *A. defectiva* infections, typically including gentamicin in combination with either ampicillin or penicillin, mirroring the approach used for enterococcal endocarditis; vancomycin alone is also considered a viable alternative [13]. Similarly, the 2023 European Society of Cardiology (ESC) Guidelines for Infective Endocarditis recommend a six-week regimen of penicillin G, ceftriaxone, or vancomycin, combined with aminoglycoside, for at least the first two weeks in the case of prosthetic valve endocarditis [14]. A study by Alberti et al. reported no vancomycin resistance and low aminoglycoside resistance among isolates of *A. defectiva*, *Granulicatella adiacens*, and *Granulicatella elegans*. Penicillin susceptibility was variable, with 53% of isolates showing intermediate susceptibility (MIC 0.25–2 µg/mL). *Granulicatella* species were generally more susceptible to penicillin than *A. defectiva*, while the latter showed greater susceptibility to ceftriaxone with variable rates of penicillin resistance [3]. The study of Cañas et al. confirms high susceptibility of *A. defectiva* strains to ceftriaxone. Moreover, all included strains were susceptible to vancomycin, although the bactericidal activity of this antibiotic might be limited by the detection of tolerance in vitro. Regarding newer antimicrobials, high levels of baseline resistance to daptomycin have been shown among *Granulicatella* and *A. defectiva* clinical isolates, limiting their use as monotherapy. Furthermore, resistance to daptomycin may develop rapidly even when susceptible at baseline and even when used in combination with ampicillin, gentamicin, or cephalosporins [6]. In the study of Prasidthrathsint et al. involving 599 NVS isolates (152 *A. defectiva* and 447 *Granulicatella* spp.), only 14% of *A. defectiva* isolates were penicillin-susceptible (21/152), although 97% of penicillin-resistant strains remained susceptible to ceftriaxone [15].

In our case, due to the unavailability of both *A. defectiva* antibiogram and the resistance genotype, vancomycin was empirically selected as the most reasonable treatment option. Considering the concomitant presence of prosthetic joint infection (PJI) and infective endocarditis, along with the absence of microbial growth for susceptibility testing, a more aggressive therapeutic strategy was adopted. This included broad-spectrum coverage targeting Gram-positive bacteria in consideration of potential β-lactam resistance or an undetected polymicrobial infection. Vancomycin was administered via continuous infusion, supported by therapeutic drug monitoring, which allowed for optimized pharmacokinetics, improved penetration into bone and valvular tissue, and reduced risk of toxicity. Additionally, a two-stage prosthetic arthroplasty was undertaken to reduce microbial burden and eradicate biofilm formation on the infected implant. Although the role of rifampin remains undefined in NVS non-endocardial pathology, in our review, its use has been reported in 5 out of 22 cases, which had a favorable outcome. However, concerns regarding polypharmacy and potential drug–drug interactions often limit its practicality in combination therapy, as in our clinical scenario. Similarly, prolonged combination therapy with an aminoglycoside was avoided due to the associated risk of nephrotoxicity.

Our case posed several challenges. Unlike most reported cases where a dental source was suspected, our patient had no history of recent odontogenic procedures, and the primary source of bacteremia remained unclear, though a prior urinary tract infection was documented. Secondly, in the absence of antimicrobial susceptibility data, treatment was guided by the most reasonable empiric option, balancing literature-based susceptibility profiles with the need for broad-spectrum coverage. In addition, the concomitant presence of endocarditis and prosthetic joint infection presented a therapeutic dilemma regarding the prioritization of intervention. To date, the only other published case of concomitant endocarditis and PJI caused by *Granulicatella* was reported by Renz et al., in which both conditions were managed conservatively, with the DAIR approach being used for PJI [5]. In contrast, due to the inability to clearly establish the source and the interval between the primary focus of bacteremia and the onset of prosthetic joint infection, our patient underwent a two-stage exchange arthroplasty.

Our review has several limitations. First of all, the analysis is based exclusively on case reports, which inherently limits the reliability and generalizability of the findings. The reported outcomes and their correlation with variables such as antimicrobial regimen, treatment duration, and surgical strategy may be confounded by inconsistent or unreported follow-up periods. Moreover, the small sample size substantially limits statistical power. Nevertheless, there appears to be a trend suggesting improved outcomes with prolonged antibiotic therapy. Further studies with larger cohorts are warranted to better understand and define optimal management strategies for PJIs caused by *A. defectiva* and *Granulicatella* spp.

## 5. Conclusions

Our case illustrates a complex presentation of *A. defectiva* infection accompanied with a literature review on prosthetic joint infections caused by *A. defectiva* and *Granulicatella* spp. Although the available evidence is primarily derived from case reports—limiting its overall reliability—there appears to be an association between prolonged antibiotic treatment and improved clinical outcomes. This may reflect the fastidious nature of these organisms, which can contribute to persistent infection if not adequately treated.

Advances in microbiological diagnostics, including MALDI-TOF mass spectrometry and molecular techniques such as 16S rRNA gene PCR, have significantly enhanced the identification of fastidious pathogens. These technologies have facilitated the recognition of emerging microorganisms that were previously underdiagnosed, potentially reshaping the epidemiology of traditionally well-characterized infections such as prosthetic joint infections and endocarditis. Sharing clinical experiences in this field is crucial for improving our understanding of pathogen behavior and refining management strategies.

## Data Availability

The original contributions presented in this paper are included in the article (Appendix A). Further inquiries can be directed to the corresponding author.

## Abbreviations

The following abbreviations are used in this manuscript:

NVS	Nutritionally variant streptococci
PJI	Prosthetic joint infection

## Appendix A

**Table A1:** A summary of the literature on Granulicatella and Abiotrophia PJI.

Reference	Age	Sex	Comorbidity	Possible Risk Factor	Symptoms	Infected Joint	Bacteria	Endocarditis	Identification	Antibiogram	Pen-S
Seguiti C et al. (our case, 2025); case report and literature review	68	M	diabetes; Barlow syndrome	urinary infection	fever; swelling	Prosthetic Knee	Abiotrophia defectiva	Yes (NVE mitral valve)	MALDI-TOF	No	NA
Ince A et al. (2002); case report [16]	65	F	alcoholism; diabetes; nephrectomy	NA	joint pain; swelling	Prosthetic Knee	Abiotrophia defectiva	No	16S rRNA	No	NA
Cassir N et al. (2011); case report [17]	71	M	NA	dental procedure	swelling	Prosthetic Knee	Abiotrophia defectiva	No	16S rRNA	Yes	Yes
Rozemejer W et al. (2011); case report [18]	71	F	polymialgia rheumatica	NA	joint pain	Prosthetic Hip	Abiotrophia defectiva	No	16S rRNA	Yes	Yes
Mougari F et al. (2013); case report [19]	55	M	NA	dental procedure	joint pain; swelling	Prosthetic Knee	Granulicatella adiacens	No	16S rRNA	Yes	Yes
Renz N et al. (2016); case report [5]	69	M	NA	dental procedure	fever; joint pain	Prosthetic Hip	Granulicatella para-adiacens	Yes (NVE mitral valve)	Culture + 16S rRNA	Yes	Intermediate
Aweid O et al. (2016); case report [20]	81	M	NA	dental procedure	fever; swelling	Prosthetic Hip	Granulicatella adiacens	No	MALDI-TOF	Yes	Yes
Pingili C et al. (2017); case report [21]	64	M	NA	dental procedure	swelling	Prosthetic Knee	Granulicatella adiacens	No	16S rRNA	No	NA
Quénard F et al. (2017); case series (3 cases) [22]	75	M	hypertension; ankylosing spondylitis	dental procedure	fistula	Prosthetic Hip	Granulicatella adiacens	No	16S rRNA	No	NA
	65	M	alcoholism; psoriasis	NA	joint pain	Prosthetic Knee	Granulicatella adiacens	No	MALDI-TOF	No	NA
	44	F	congenital hip dysplasia	surgical resection of cystic lesion	joint pain; fistula	Prosthetic Hip	Granulicatella adiacens	No	MALDI-TOF + 16S rRNA	No	NA
Mavrommati A B et al. (2017); case report [23]	76	F	NA	NA	joint pain	Prosthetic Knee	Granulicatella adiacens	No	Vitek 2	Yes	NA
Tooley T R et al. (2019); case report [24]	74	M	cardiopathy; dysphagia; past PJI	NA	joint pain	Prosthetic Knee	Abiotrophia defectiva	No	MALDI-TOF	No	NA
Wan J et al. (2020); case report [25]	65	M	diabetes; hypertension; CKD	NA	joint pain; swelling	Prosthetic Knee	Abiotrophia defectiva	No	Culture	No	NA
Van Someren M W M et al. (2020); case report [26]	61	M	NA	NA	joint pain	Prosthetic Hip	Abiotrophia defectiva	No	Culture	Yes	No
Kocazeybek E et al. (2020); case report and literature review [27]	69	F	NA	dental procedure	joint pain; swelling	Prosthetic Knee	Abiotrophia defectiva	No	MALDI-TOF	Yes	Yes
Badrick T C et al. (2021); case report and literature review [28]	79	M	coronaropathy	dental procedure	joint pain	Prosthetic Hip	Granulicatella adiacens	No	16S rRNA	No	NA
Narayana Murthy S et al. (2021); case report [29]	65	M	NA	acupuncture	joint pain; swelling	Prosthetic Knee	Granulicatella adiacens	No	Culture	No	NA
Young J N et al. (2022); case report [30]	71	M	Charcot–Marie–Tooth	left foot cellulitis; dental procedure	swelling	Prosthetic Knee	Abiotrophia defectiva	No	16S rRNA	No	NA
Quirino A et al. (2022); case report and literature review [31]	69	F	NA	previous Staphylococcus warneri PJI	joint pain; swelling	Prosthetic Knee	Abiotrophia defectiva	No	MALDI-TOF; Vitek 2; 16S rRNA	Yes	Yes
Nooh A et al. (2025); case report [32]	66	F	hypertension; hypotiroidism; osteoporosis	urinary infection	joint pain; swelling	Prosthetic Knee	Abiotrophia defectiva	No	Culture	No	NA
Lovering E et al. (2025); case report [33]	66	F	prosthetic aortic valve	past Granulicatella adiacens bacteremia	swelling	Prosthetic Knee	Granulicatella adiacens	No	Vitek 2	Yes	Intermediate

NA: Not applicable (not specified).

**Table A2:** A summary of the literature on the treatment of Granulicatella and Abiotrophia PJI and outcomes.

Reference	Surgical Option	Treatment Duration (Week)	Antibiotics	Outcome
Seguiti C et al. (our case, 2025); case report and literature review	two stage	10	vancomycin	Cured
Ince A et al. (2002); case report [16]	DAIR	5	cefazolin (10 d) + ciprofloxacin	Relapse (two stage)
Cassir N et al. (2011); case report [17]	two stage	39	amoxicillin	Cured
Rozemejer W et al. (2011); case report [18]	two stage	6	penicillin	Cured
Mougari F et al. (2013); case report [19]	two stage	15	amoxicillin + rifampin	Cured
Renz N et al. (2016); case report [5]	DAIR	13	penicillin + gentamycin (4 w) then levofloxacin + rifampin	Cured
Aweid O et al. (2016); case report [20]	DAIR	6	piperacillin/tazobactam + vancomycin + fucidic acid (4 w) then meropenem + daptomycin	Died from pneumonia
Pingili C et al. (2017); case report [21]	two stage	6	ertapenem	Cured
Quénard F et al. (2017); case series (3 cases) [22]	two stage	26	amoxicillin + clindamycin	Cured
	one stage	26	clindamycin + rifampin	Cured
	DAIR	26	imipenem/cilastatin + ciprofloxacin (4 w) then amoxicillin/clavulanate + ciprofloxacin	Cured
Mavrommati A B et al. (2017); case report [23]	two stage	NA	ciprofloxacin + rifampin	Cured
Tooley T R et al. (2019); case report [24]	two stage	20	ceftriaxone (6 w) then cephalexin	Cured
Wan J et al. (2020); case report [25]	two stage	8	vancomycin (2 w) then amoxicillin + rifampin	Cured
Van Someren M W M et al. (2020); case report [26]	two stage	17	ciprofloxacin (6 w) + clindamycin (17 w)	Cured
Kocazeybek E et al. (2020); case report and literature review [27]	two stage	4	penicillin + gentamycin	Cured
Badrick T C et al. (2021); case report and literature review [28]	two stage	6	benzylpenicillin	Cured
Narayana Murthy S et al. (2021); case report [29]	DAIR	NA	vancomycin (duration not specified) then clindamycin (6 w)	Cured
Young J N et al. (2022); case report [30]	two stage	6	ceftriaxone	Reinfection (Enterobacter cloacae)
Quirino A et al. (2022); case report and literature review [31]	retention	9	ampicillin + ceftriaxone (3 w) then amoxicillin + doxycyclin	Cured
Nooh A et al. (2025); case report [32]	two stage	12	ceftriaxone + teicoplanin (6 w) then oral therapy (not specified)	Cured
Lovering E et al. (2025); case report [33]	two stage	NA	vancomycin (total duration not specified)	Cured

NA: Not applicable (not specified).

**Table A3:** Descriptive and inferential statistics of potential features related to dichotomous outcome: not cured vs. cured.

Outcome:	Not Cured (N = 3)	Cured (N = 19)	*p*-Value
**Age**			
Mean (SD)	72.3 (8.08)	66.9 (7.87)	0.361
**Sex**			
F	1 (33.3%)	7 (36.8%)	1
M	2 (66.7%)	12 (63.2%)	
**Diabetes_yes_no**			
No	1 (50.0%)	8 (80.0%)	1
Yes	1 (50.0%)	2 (20.0%)	
**Dental procedure**			
No	0 (0.0%)	6 (46.0%)	0.64
Yes	2 (100.0%)	7 (53.0%)	
**Infected joint**			
Prosthetic Hip	1 (33.3%)	6 (31.5%)	1
Prosthetic Knee	2 (66.7%)	13 (68.5%)	
**Bacteria**			
Abiotrophia defectiva	2 (66.7%)	9 (47.4%)	0.793
Granulicatella adiacens	1 (33.3%)	9 (47.4%)	
Granulicatella para-adiacens	0 (0%)	1 (5.2%)	
**Endocarditis**			
No	3 (100%)	17 (89.5%)	1
Yes (NVE mitral valve)	0 (0%)	2 (10.5%)	
**Antibiogram**			
No	2 (66.7%)	10 (52.6%)	1
Yes	1 (33.3%)	9 (47.4%)	
**Penicillin S**			
Intermediate	0 (0%)	2 (25.0%)	0.755
No	0 (0%)	1 (12.5%)	
Yes	1 (33.3%)	5 (62.5%)	
**Surgical option**			
DAIR	2 (66.7%)	3 (15.7%)	0.274
One stage	0 (0%)	1 (5.3%)	
Retention	0 (0%)	1 (5.3%)	
Two stage	1 (33.3%)	14 (73.7%)	
**Treatment duration, weeks**			
Mean (SD)	5.67 (0.577)	15.2 (9.85)	0.048
